# Advances in *Pseudostellaria heterophylla* Research: Current Status and Future Directions

**DOI:** 10.3390/molecules30173656

**Published:** 2025-09-08

**Authors:** He Li, Shiying Wang, Zisong Yang, Pengda Ma

**Affiliations:** 1 College of Resources and Environment, ABA Teachers College, Wenchuan 623002, China; 17393648665@163.com (H.L.); wsy891023@outlook.com (S.W.); 2 College of Life Sciences, Northwest A&F University, Yangling 712100, China

**Keywords:** *Pseudostellaria heterophylla*, cyclic peptides, immunomodulation, phytohormone crosstalk, sustainable utilization

## Abstract

*Pseudostellaria heterophylla*, a dual-purpose medicinal and edible herb, has shown significant pharmacological potential, particularly through its immunomodulatory and antitumor activities. This review provides insights into the phytohormone regulatory mechanisms and active-component biosynthesis, highlighting key metabolic pathways and yield-optimization strategies. The interactions between hormones and genes in root morphology and metabolite accumulation are discussed, offering new perspectives for molecular breeding. Additionally, a multidisciplinary framework is proposed to address cultivation challenges and quality enhancement, laying the groundwork for sustainable utilization of this valuable medicinal plant.

## 1. Introduction

*Pseudostellaria heterophylla* (Miq.) Pax ex Pax et Hoffm.(*P. heterophylla*), also known as haiershen, tongshen, four-leaf ginseng, and double-batch seven, belongs to the *Caryophyllaceae* family. Its dried tuberous roots are primarily distributed in provinces of China, such as Liaoning, Hebei, Shandong, Anhui, and Sichuan. *P. heterophylla* possesses the effects of invigorating qi to strengthen the spleen, promoting the production of body fluids to nourish the lungs, and is commonly used in the treatment of symptoms such as spleen deficiency with fatigue, poor appetite, post-illness weakness, and qi and yin deficiency. In traditional Chinese medicine (TCM), “qi” refers to the vital energy that maintains human physiological activities, while “yin” represents the material basis for nourishing the body (e.g., body fluids and blood). *P. heterophylla* is also indicated for spontaneous sweating, thirst, and dry cough due to lung dryness [1]. It shares similar efficacies with ginseng but is mainly characterized by “mild nourishment” that replenishes deficiencies without causing abrupt increases [2]. Currently, *P. heterophylla*-based pharmaceuticals, such as Jianwei Xiaoshi Tablets and Compound *P. heterophylla* Granules, are commercially available. Modern clinical medical research has demonstrated that *P. heterophylla* exhibits immune-enhancing [3], antitussive [4], antifatigue [5], antitumor [6], antioxidant [7], and cardioprotective effects [8], and it has also achieved breakthroughs in the treatment of asthma [9], diabetes [10] iron-deficiency anemia in children [11], anorexia [12], gastric cancer [13], and other diseases. Based on current chemical research, various components have been identified in *P. heterophylla*, including cyclic peptides, polysaccharides, amino acids, saponins, and sapogenins [14]. In recent years, wild *P. heterophylla* resources have decreased annually, with the main source now being artificial cultivation.

However, in the process of artificial cultivation of *P. heterophylla*, the problem of continuous cropping obstacles is particularly prominent—long-term continuous cropping not only leads to the deterioration of soil physical and chemical properties and the imbalance of microbial communities, but also directly causes a significant reduction in the biomass of underground tubers and a sharp decline in quality, seriously undermining its medicinal and economic value [14]; at the same time, replantation diseases induced by continuous cropping further exacerbate this dilemma, pushing the biomass and quality of *P. heterophylla* tubers into a state of continuous decline [15]. With the continuous advancement of the modernization of traditional Chinese medicine, as well as the gradual deepening of public understanding of Chinese medicinal materials and the increasing awareness of health care, the market demand for high-quality *P. heterophylla* is constantly rising. Nevertheless, the current *P. heterophylla* industry still faces multiple challenges, such as insufficient optimization of cultivation techniques, unclear quality regulation mechanisms, and limited sustainable resource supply. How to solve these problems through technological innovation and promote *P. heterophylla* to better meet modern medical needs remains a task requiring in-depth research on many key scientific issues. The main objective of this review is to systematically summarize the current research progress of *P. heterophylla* (including active components, pharmacological mechanisms, growth regulation, and cultivation challenges) and propose future directions, thereby providing a theoretical basis for its sustainable utilization and industrial development.

## 2. Research Progress on Active Components of *Pseudostellaria heterophylla*

To summarize the research progress on the chemical bioactive constituents of *P. heterophylla*, a literature search was conducted on databases such as PubMed, Web of Science, CNKI, and Wanfang Data. The search terms comprised “*Pseudostellaria heterophylla*”, “*Radix Pseudostelariae*”, “bioactive constituents”, “cyclic peptides”, “polysaccharides”, and “pharmacological activities”. The summary revealed that the chemical constituents of *P. heterophylla* have been elucidated in recent years (Table 1). To date, a total of 19 cyclic peptide compounds have been isolated from *P. heterophylla*, including 9 heterophyllins (A–H and J) and 10 pseudostellarins (A–H, K, and L) [16]. These cyclic peptides are mainly isolated from the dried tuberous roots and fibrous roots of *P. heterophylla*: high-performance liquid chromatography (HPLC) is mainly used for isolation, while nuclear magnetic resonance (NMR, e.g., ^1^H-NMR and ^13^C-NMR) and electrospray ionization–mass spectrometry (ESI-MS) are applied for structural identification—these techniques help clarify the molecular structures of the compounds and verify the purity of the components [16,17,18]. In addition, four sapogenins and six saponins (e.g., pseudotellarinoside A and acutifoliside D) have also been isolated from *P. heterophylla*: silica gel chromatography is used for preliminary separation, semi-preparative HPLC for purification, and mass spectrometry (MS) for structural identification [17,19].

As important active components of *P. heterophylla*, polysaccharides account for 18.38–64.95% of its content, and their composition is affected by geographical origin, harvest time, and cultivation management. A total of 10 polysaccharides have been isolated, and 7 oligosaccharides have been identified—including 2 pairs of isomers (DP 3-1/DP 3-2 with a molecular weight of 503.2 Da; DP 4-1/DP 4-2 with a molecular weight of 665.2 Da) and 3 branched oligosaccharides (DP 5, DP 6, and DP 7 with molecular weights of 827.3, 989.3, and 1151.4 Da, respectively) [16,20]. The qualitative and quantitative analysis of the aforementioned carbohydrate components is achieved by an ultra-high performance liquid chromatography-charged aerosol detector (UPLC-CAD) and ultra-high performance liquid chromatography–quadrupole time-of-flight mass spectrometry (UPLC-QTOF MS) [20].

**Figure 1:**
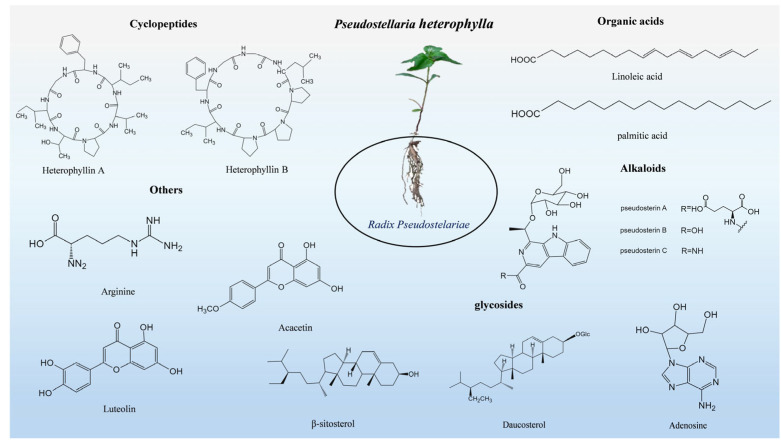
Structures of main chemicals in *Pseudostellaria heterophylla*.

Besides cyclic peptides, polysaccharides, and saponins, *P. heterophylla* also contains volatile oils (e.g., furfuryl alcohol and palmitic acid), sterols (e.g., daucosterol, Δ^7^-stigmasten-3β-ol, and Δ^7^-stigmasten-3-O-β-D-glucopyranoside), and seven essential amino acids for humans [19,21]. Among them, volatile oils are identified by gas chromatography daucosterol mass spectrometry (GC-MS), while sterols are confirmed by thin-layer chromatography (TLC) and MS [17,22]. Pseudosterols A–C (1–3) isolated from the tuberous roots of *P. heterophylla* possess a 1-ethyl-3-formyl-β-carboline structure and exhibit cardioprotective activity; their structures are elucidated by NMR (including HSQC and HMBC) and quantum chemical calculations [19] (Table 2 and Figure 1).

Although the medicinal value of *P. heterophylla* has been widely recognized in traditional Chinese medicine, its mechanism of action remains unclear. Given its characteristic “multi-component and multi-target” properties typical of traditional Chinese medicines, elucidating the functions and pathways of its active components is crucial for a comprehensive understanding of its disease-resistance mechanisms.

**Figure 2:**
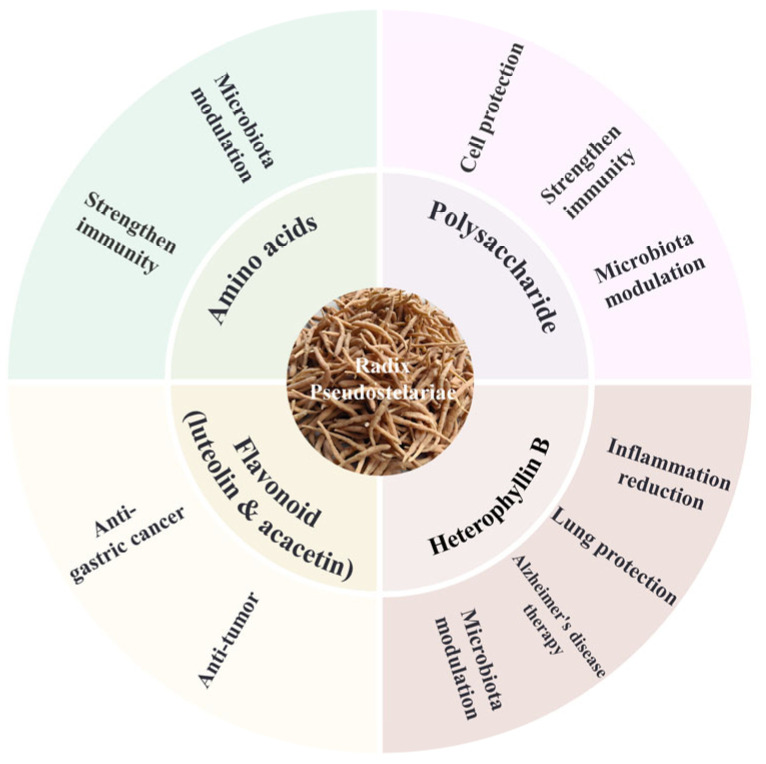
The main medicinal ingredients and corresponding effects of *Pseudostellaria heterophylla*.

The diverse active components of *P. heterophylla* exhibit extensive pharmacological effects, which have been validated through in vitro and in vivo studies (Figure 2). Zhang et al. found that, in mice, treatment with the superfine powder suspension of *P. heterophylla* enhanced the levels of immune factors such as IL-2 and IFN-γ, which are critical for immune cell development, thereby boosting immune function. Meanwhile, it reshaped the gut microbiota by increasing the abundance of beneficial bacteria (e.g., *Akkermansia*, *Roseburia*, unclassified *Clostridiaceae*, *Mucispirillum*, *Anaeroplasma*, and *Parabacteroides*) and reducing the abundance of pathogenic bacteria (e.g., *Cupriavidus* and *Staphylococcus*). These microbial changes stimulated the production of beneficial metabolites, with the bacteria–host metabolic network primarily involved in amino acid and carbohydrate metabolism, suggesting that amino acids and heterophyllin B in *P. heterophylla* may be key regulators of the gut microbiota [22].

Li et al. optimized the extraction process of hydrophilic polysaccharides (PHPs) from *P. heterophylla* using single-factor experiments and orthogonal design, and explored their properties under ultrasonic treatment. They found that these polysaccharides (especially after 500 W ultrasonic treatment) enhanced the proliferation (maximum proliferation rate of 33.62% at 250 μg/mL) and phagocytic activity of macrophage RAW264.7, increased NO secretion and iNOS expression, and significantly improved antioxidant capacity (enhanced DPPH and hydroxyl radical scavenging) and immune function (elevated white blood cell/lymphocyte counts and spleen index in mice) [22]. Wang Quanxi’s team, focusing on developing efficient, non-toxic, and safe traditional Chinese medicine immune adjuvants, demonstrated that oral administration of *P. heterophylla* fibrous root polysaccharide (RPFRP, not PHP) promoted splenic lymphocyte proliferation (significantly increasing T cell stimulation index), enhanced NK cell cytotoxic activity (high-dose group showed the best trend), and upregulated the transcription level of IL-2 mRNA in mouse spleens; IL-4, IL-10, and IFN-γ mRNA showed an upward trend but no significant difference compared to the control [23]. Further metabolomic and microbiomic analyses by the same team revealed that *P. heterophylla* polysaccharide (RPP, not PHP) increased the abundance of beneficial bacteria (e.g., *Prevotella copri*) in feces and decreased potential pathogens (e.g., *Paraeggerthella*) [24]. Notably, *Prevotella copri* elevated taurodeoxycholic acid (TDCA) levels, which exerted immunoregulatory effects through multiple pathways: promoting proliferation and accelerating the cell cycle of normal gastric epithelial cells (GES-1) via activating the IL-6/JAK1/STAT3 pathway; inducing IL-8 gene expression in colonic epithelial cells through RelA phosphorylation (this effect could be blocked by an IKKβ inhibitor) [25,26]; specifically, TDCA induces IL-8 expression in HT-29 cells and primary colonic epithelial cells in a dose- and time-dependent manner, relying on IKKβ-mediated RelA phosphorylation (not classical IκB degradation) to drive IL-8 transcription [27]; and ameliorating inflammation by inhibiting the priming phase of NLRP3 inflammasome (via the GPCR19-cAMP-PKA-NF-κB axis) and suppressing the activation phase (via enhancing GPCR19-P2X7R colocalization to inhibit Ca^2+^ mobilization and NLRP3-ASC oligomerization) [28].

Heterophyllin B, a representative cyclic peptide in *P. heterophylla*, regulates pathological features of Alzheimer’s disease (AD), splenic immune function, and gut microbiota composition via the spleen–gut–brain axis, ultimately alleviating AD-induced cognitive impairment. Specifically, it reduces neuroinflammation, restores synaptic loss, decreases extracellular β-amyloid (Aβ) and Tau protein expression, and downregulates the splenic Th1/Th2 ratio [29]. It can also penetrate the blood–brain barrier, inhibit Aβ-induced neuronal apoptosis, promote neurite regeneration (increasing the density of β3-tubulin and MAP2-positive neurites), and significantly improve recognition and spatial memory deficits in AD mice [30]. In a bleomycin (BLM)-induced pulmonary fibrosis (PF) mouse model, Wen et al. found that heterophyllin B inhibited fibroblast transformation by activating AMPK and suppressing TGF-β, reduced extracellular matrix (ECM) deposition by downregulating COL-1 and α-SMA, and decreased STING expression. Notably, the AMPK inhibitor Compound C reversed these effects, suggesting it protects against BLM-induced PF by inhibiting the TGF-Smad2/3 pathway and AMPK-mediated STING signaling [31].

**Figure 3:**
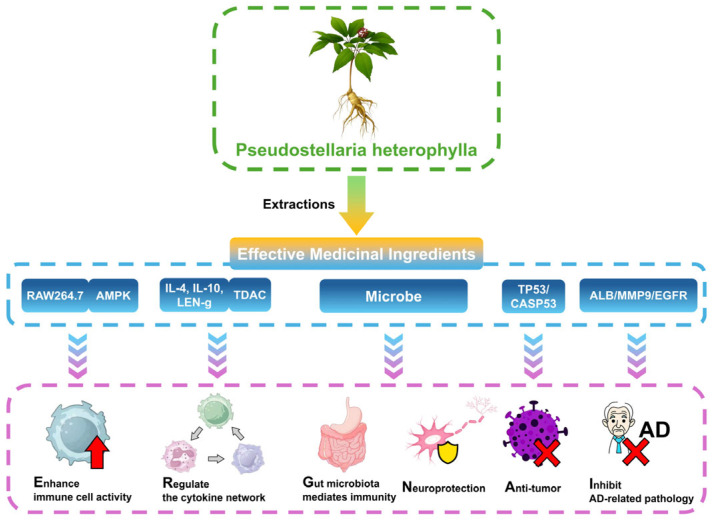
Immunomodulation and neuroprotective mechanisms of *Pseudostellaria heterophylla*: mediated effects based on multi-target signaling pathways and intestinal microbiota.

Network pharmacology studies have further predicted the targets and pathways of *P. heterophylla* components. For example, constructing an interaction network between heterophyllin B and AD identified five key targets (ALB, MMP9, Src, EGFR, and MMP2) closely related to its therapeutic effect on AD [30]. Additionally, luteolin and acacetin, active components of *P. heterophylla*, specifically bind to TP53 in gastric cancer (GC) cells, promote its phosphorylation, and inhibit GC cell viability via ROS- and P53-related signaling pathways [13]. Moreover, active components may affect diffuse large B-cell lymphoma (DLBCL) by targeting CASP3 [6] (Table 3 and Figure 3).

Beyond investigating the pharmacological mechanisms of known active components, it is essential to dissect the biosynthetic pathways of these medicinal constituents at the molecular level, with the ultimate goal of enhancing their production via biotechnological approaches. For example, Lin et al. successfully cloned two key enzyme genes involved in saponin biosynthesis—3-hydroxy-3-methylglutaryl-coenzyme A reductase (*HMGR*) and squalene epoxidase 1 (*SQE1*)—from the tubers of *P. heterophylla* using RT-PCR combined with rapid amplification of cDNA ends (RACE) technology. To validate their functions, recombinant expression vectors (e.g., *pRI 101-AN*) were constructed and introduced into *Nicotiana tabacum* cv. K326 via Agrobacterium-mediated leaf disc transformation. Subsequent experiments, including qPCR analysis of gene transcription levels, enzyme activity assays, and spectrophotometric determination of total triterpene content, confirmed that heterologous expression of *PhHMGR* and *PhSQE1* significantly increased total triterpene content in transgenic tobacco, clarifying their regulatory roles in the mevalonate (MVA) pathway [30,32].

Similarly, Lu et al. focused on asparaginyl endopeptidases (AEPs)—enzymes that catalyze the cyclization of linear peptides by recognizing asparagine/aspartate (Asn/Asp) residues at the C-terminus of precursor peptides. By systematically mining *P. heterophylla* transcriptome data and designing specific primers based on conserved AEP sequences, they cloned the *PhAEP* gene. For functional validation, a transient co-expression system in *Nicotiana benthamiana* was employed: the *PrePhHA* gene (encoding the linear precursor of cyclic peptide HA) and *PhAEP* were introduced into tobacco leaves via Agrobacterium infiltration. Ultra-performance liquid chromatography–electrospray ionization-time-of-flight mass spectrometry (UPLC-ESI-TOF MS/MS) analysis of leaf extracts showed that cyclic peptide HA was only detectable in the co-expression group, directly confirming the mediating role of PhAEP in peptide cyclization [33]. These studies provide a paradigm for dissecting the biosynthetic pathways of active components in *P. heterophylla*, integrating techniques such as molecular cloning, heterologous expression, and metabolomic analysis. Future research can leverage these identified key enzymes (HMGR, SQE1, and PhAEP) as molecular targets to conduct metabolic engineering studies, aiming to increase the production of high-value components like triterpenoid saponins and cyclic peptides. Integrating multi-omics data (transcriptomics and metabolomics) with enzyme kinetic analyses may further reveal the cross-regulation between saponin and cyclic peptide biosynthetic pathways, optimizing metabolic flux. Additionally, combining synthetic biology tools (e.g., CRISPR-Cas9) with heterologous expression systems (tobacco and microbial chassis) is expected to overcome the limitations of natural production and enable large-scale synthesis. Finally, translating these molecular-level findings into standardized cultivation practices and clinical applications—by correlating gene expression profiles with medicinal quality—will bridge the gap between basic research and industrial/therapeutic applications, fully exploiting the medicinal value of *P. heterophylla*.

*P. heterophylla* possesses a rich diversity of bioactive constituents, including cyclic peptides, polysaccharides, and flavonoids, which collectively confer it with promising pharmacological potential in immune regulation, antitumor activity, and neuroprotection. These effects are achieved through complex interactions involving gut microbiota modulation, metabolite generation, and the activation of specific signaling pathways. Nevertheless, critical knowledge gaps remain: the synergistic mechanisms among multiple components are not yet clear, and the biological activities of some understudied constituents (e.g., certain oligosaccharides and volatile oils) have not been systematically characterized. To address these issues, future research should prioritize in-depth exploration of component-specific functions, validation of key molecular targets, and advancement of clinical translation—while overcoming technical challenges such as compound isolation and multi-omics data integration.

## 3. Studies on the Growth and Development of *Pseudostellaria heterophylla*

Post-COVID-19, with the continuous enhancement of global health awareness and the ongoing promotion of the “homology of medicine and food” concept, the demand for traditional Chinese medicinal materials has surged significantly. Among these materials, *P. heterophylla* has attracted considerable attention due to its dual effects of tonifying Qi, strengthening the spleen, and moistening the lungs. As a key medicinal herb, its wild resources have been severely depleted due to overharvesting, making artificial high-yield cultivation the core approach to meet market demand [21]. However, the cultivation of *P. heterophylla* is confronted with a series of intertwined challenges: tuberous root propagation, the dominant propagation method, is susceptible to viral infections; the deterioration of soil physicochemical properties (such as acidification and nutrient sequestration) exacerbates the decline in quality; continuous cropping, driven by the combined effects of microbial community dysbiosis and allelopathy, leads to a 15–90% yield loss; and environmental stresses, including drought and salinity, further impair productivity. These issues not only cause substantial economic losses to farmers but also hinder the sustainable development of the *P. heterophylla* industry.

Notably, the yield and quality of *P. heterophylla* are not regulated by isolated factors but by the synergistic interactions between biological and abiotic elements. For instance, viral accumulation in tuberous roots can be exacerbated by soil acidification induced by continuous cropping; meanwhile, phytohormones such as abscisic acid (ABA) and gibberellin (*GA*_3_), as well as key gene families, including *NF-Y* and *GST*, further mediate tuber development and the accumulation of bioactive components (e.g., polysaccharides, saponins, and cyclic peptides) under such growth conditions. Additionally, changes in soil nutrient availability interact with stress resistance mechanisms, affecting the plant’s tolerance to drought and salinity. This section synthesizes recent research findings, systematically clarifies the mechanisms of key influencing factors (including propagation systems, soil microenvironment, continuous cropping dynamics, stress resistance mechanisms, and phytohormone regulation); compares differences in research conclusions; and explores areas requiring further in-depth investigation (such as the molecular mechanism of multi-virus synergistic infection, the long-term field effects of phosphorus-modified biochar, and the regulatory network among stress-responsive genes). It aims to provide a comprehensive and systematic theoretical basis for the efficient cultivation of *P. heterophylla*.

### 3.1. Factors Affecting Pseudostellaria heterophylla Yield and Yield-Increasing Strategies

**Figure 4:**
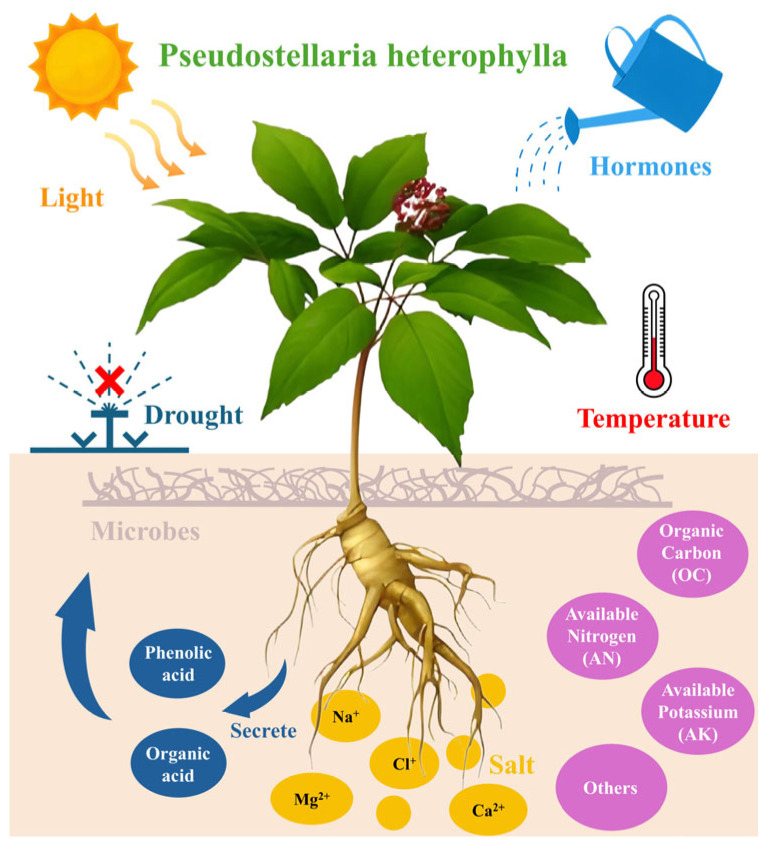
Factors influencing the yield/quality of *Pseudostellaria heterophylla*.

The yield and quality of *P. heterophylla* are jointly determined by propagation systems, soil microenvironments, continuous cropping dynamics, and stress resistance mechanisms. These factors interact closely: for instance, virus-free propagation materials (from optimized propagation methods) can only exert their full potential in soils with balanced physicochemical properties, while soil degradation (a driver of continuous cropping obstacles) further weakens the plant’s ability to resist viruses and stresses. The following sections analyze each factor and their interactions (Figure 4).

#### 3.1.1. Reproduction Methods and Virus Prevention and Control

Propagation of *P. heterophylla* primarily includes sexual reproduction via seeds and asexual reproduction through tuberous roots. Sexual reproduction is rarely used in commercial production due to low germination rates (<30%) and high genetic variation (>15%), which lead to inconsistent seedling quality [21]. In contrast, tuberous root propagation is dominant for its simplicity and trait stability; however, this method is highly susceptible to viral infections. Studies have shown that viruses mainly spread through infected tuberous roots (the primary propagation material) and vector insects (e.g., aphids) [34]. Four viruses have been confirmed to infect *P. heterophylla*: Turnip Mosaic Virus (TuMV), Broad Bean Wilt Virus (BBWV), Cucumber Mosaic Virus (CMV), and Tobacco Mosaic Virus (TMV). Among these, TuMV and BBWV are the most prevalent in major producing areas (e.g., Zherong, Fujian), with TuMV showing a 100% detection rate in the surveyed samples of this region [35].

To address viral issues, two biotechnological detoxification methods have been widely studied, each with its own characteristics. Low-temperature treatment combined with microstem tip culture (2–4 °C for 60–90 days, followed by 0.2 mm microstem tip excision) achieves a survival rate of 64.7%, with BBWV and TuMV detoxification rates of 63.6% and 31.8%, respectively. This method maintains genetic stability (critical for preserving elite cultivars like ‘Zheshen 1’), but its effectiveness in eliminating TuMV is relatively limited, which may be related to the virus’s strong infectivity and distribution in near-meristematic regions. On the other hand, embryo culture achieves complete detoxification for both BBWV and TuMV, with a survival rate of 56.7%, yet there is a need to further explore its potential impact on genetic traits—such as whether it may alter key characteristics like tuber size and active component content, which is relevant for its large-scale application [36]. Currently, most studies focus on detoxification efficiency in laboratory settings, and there is a need to collect more field performance data (e.g., long-term yield and quality) of detoxified seedlings from these two methods to better guide practical production. Furthermore, Yang et al. constructed a TuMV-ZR expression vector and confirmed that viruses accumulate preferentially in tuberous roots, identifying tubers as the core carrier for viral transmission [37]. However, existing research has not yet fully clarified the mechanism of synergistic infection by multiple viruses (e.g., whether TuMV and BBWV colocalize in tubers to enhance pathogenicity) or developed broad-spectrum antiviral strategies suitable for large-scale cultivation.

In summary, the choice of propagation method requires considering detoxification efficiency, genetic stability, and field adaptability. Future work could focus on quantifying the genetic variation associated with embryo culture and evaluating how detoxified seedlings perform in different soil conditions to better connect laboratory research with agricultural practice.

#### 3.1.2. Soil Environment and Nutrient Regulation

Soil physicochemical properties directly determine the synthesis of *P. heterophylla*’s active components (polysaccharides and saponins) and the health of propagated tubers, and existing studies have observed some variations in results that warrant further exploration, such as the optimal soil pH for quality improvement and the long-term performance of soil amendments.

Regarding soil pH, Zheng et al. investigated 22 sampling sites in Guizhou and found that soil pH < 5 increased the availability of organic carbon (OC), available nitrogen (AN), and available potassium (AK), thereby enhancing polysaccharide content by 9.8%. However, the same study also reported that soil pH > 7 improved water-soluble extractives and inorganic elements (Mg and Ca)—traits that are important for the medicinal quality of *P. heterophylla* [38]. This suggests that the effect of pH is mediated by nutrient availability rather than direct physiological regulation, and there is no one-size-fits-all pH threshold that applies to all production goals, especially since few studies have simultaneously considered yield, polysaccharide content, and water-soluble extractives.

Soil amendments, particularly phosphorus-modified biochar, have shown potential in optimizing the rhizosphere environment. Ng et al. reported that applying 5% phosphorus-modified biochar (by mass) reduced soil-available Cd by 73.0% and Cl by 49.3%, while increasing tuber polysaccharide content by 78.8% and saponin content by 27.8% [39]. This is attributed to the modified biochar’s increased specific surface area (observed via SEM), enhanced adsorption capacity for toxic elements, and slow release of phosphorus. In contrast, unmodified peanut shell biochar at 5% inhibited tuber growth (yield reduced by 40.4%) due to increased soil osmotic suction (440.2 kPa) and Cl accumulation [39]. Most existing studies on biochar focus on short-term (4-month) effects, and there is a need to conduct long-term field trials (covering three or more cropping cycles) to verify whether phosphorus-modified biochar maintains its effectiveness over time or causes nutrient imbalances (e.g., excessive P fixation).

Another area worth exploring is the dynamic relationship between soil nutrients and active components. Zheng et al. found that water-soluble extractives (a key active component indicator of *Radix Pseudostellariae*) were positively correlated with available nitrogen (AN) in Guizhou planting areas, while polysaccharides (the core active component) showed a negative correlation with available potassium (AK), indicating divergent responses of different active components to soil nutrients [38]. Additionally, Mo et al. reported that calcium and magnesium deficiency could reduce the accumulation of water-soluble substances but increase polysaccharides and total ash in *P. heterophylla*, further suggesting that nutrient effects on active components are component-specific [40]. This does not point to a dose-dependent effect of AN, but rather reflects that soil nutrients (e.g., AN, AK, Ca, and Mg) act differently on various active components of *P. heterophylla.* Currently, no quantitative model connects specific nutrient levels to active component synthesis, which limits precise fertilization guidance for its cultivation.

As we move to the next challenge, while optimizing soil physicochemical properties creates a favorable foundation for *P. heterophylla* growth, long-term monoculture disrupts this balance by altering soil microbial communities and accumulating toxic root exudates—leading to continuous cropping obstacles. The following section explores how these soil-related changes interact with biological factors to exacerbate yield loss.

#### 3.1.3. Synergistic Mechanism and Comprehensive Prevention and Control of Continuous Cropping Obstacles

Continuous cropping of *P. heterophylla* leads to significant yield reduction (e.g., yield decreasing to 70% of the newly planted after 2-year monoculture), primarily driven by three synergistic factors: soil degradation, microbial community dysbiosis, and allelopathy [41], with recent reviews also confirming these factors as core drivers of continuous cropping obstacles in *P. heterophylla* [42]. Existing studies have identified these individual factors, but there is room to deepen our understanding of their interactions to develop more comprehensive prevention strategies.

Soil degradation under continuous cropping is characterized by “nutrient sequestration” rather than simple deficiency. Jiao et al. found that after years of monoculture, soil total phosphorus (P) and available potassium (AK) contents were significantly higher than those in newly planted soil, but their bioavailability decreased possibly due to complexation with root exudates. This sequestration is further exacerbated by soil acidification (soil pH in continuous cropping is lower than that in newly planted soil), which reduces the availability of P and calcium (Ca) [41]. However, the exact relationship between acidification and nutrient sequestration—whether acidification drives nutrient sequestration or vice versa—requires further clarification.

Microbial community dysbiosis is a key driver of disease outbreaks. High-throughput sequencing showed that long-term monoculture (3 years) significantly reduced the abundance of antagonistic microorganisms such as *Trichoderma* spp., while increasing the abundance of pathogenic *Fusarium oxysporum* [43,44]. Notably, three *Pseudomonas palleroniana* strains (B-BH16-1, B-JK4-1, and HP-YBB-1B) isolated from the rhizosphere of diseased *P. heterophylla* exhibit broad-spectrum antifungal activity against 11 dominant pathogens of this plant (including *F. oxysporum*, *Alternaria alternata*, and *Botryotinia fuckeliana*), with inhibition rates ranging from 3.71% (against *F. tricinctum*) to 56.55% (against *B. fuckeliana*); their antifungal effects are attributed to divergent secondary metabolites (tolaasin I/tolaasin F, sessilin A, and putisolvin) and carbohydrate-active enzymes (CAZymes) like chitinase and lysozyme that disrupt pathogen cell walls [45]. Moreover, certain biocontrol agents have been found to suppress specific soil-borne pathogens of *P. heterophylla* through targeted antagonism, providing additional options for mitigating microbial dysbiosis [46]. This shift is mediated by root exudates, particularly phenolic acids: in continuous cropping of *P. heterophylla*, phenolic acids (e.g., vanillin) can promote the proliferation of pathogenic microorganisms such as *Kosakonia sacchari*—vanillin enhances glycolysis/gluconeogenesis, fatty acid biosynthesis, and bacterial chemotaxis in *K. sacchari*—while *K. sacchari* further converts vanillin to protocatechuic acid, which inhibits the growth and biofilm formation of beneficial *Bacillus pumilus* by suppressing its citrate cycle and novobiocin biosynthesis [15,47]. Additionally, a new leaf blight disease of *P. heterophylla* caused by *Epicoccum sorghinum* has been reported in Tongren, Guizhou, with a field incidence exceeding 70%; this pathogen forms pink concentric colonies on PDA and produces chlamydospores (4.5–27.4 × 5.7–36.2 μm), further expanding the spectrum of diseases threatening *P. heterophylla* cultivation [48]. While this “pathogen enrichment–beneficial microbe suppression” loop is known to exacerbate disease risk, the specific molecular mechanisms by which phenolic acids induce these microbial metabolic changes still need to be fully elucidated.

Current prevention strategies have limitations when used alone. Biological control with *Bacillus thuringiensis* suppresses *F. oxysporum* growth but does not restore soil nutrient availability [45]; protease inhibitors (PHTI) disrupt pathogen cell membranes but have no significant impact on improving microbial community structure [47]. In contrast, the “flooding + microbial fertilizer” (RP-WF-BF) treatment achieves a synergistic effect: it increases the abundance of beneficial *Burkholderia* by 48.4%, enhances the expression of nitrogen cycling genes (AOB by 9.31-fold, nosZ by 1.24-fold), and restores tuber yield to 90% of that in the first cropping [41]. However, this strategy has only been validated in red soils in Fujian and needs to be tested and optimized in other soil types (e.g., yellow-brown soils in Guizhou) to expand its applicability.

Wild *P. heterophylla* resists diseases through high soil microbial diversity, indicating that artificial cultivation systems lack the ecological resilience of natural ecosystems [47]. Currently, only 5–8 beneficial strains have been isolated, which is far fewer than the diverse microbial communities present in wild habitats. Thus, further exploration and isolation of key functional microorganisms from the rhizosphere of wild *P. heterophylla* remain important tasks for future research.

### 3.2. Regulation Mechanism of Phytohormones on Growth, Development, and Quality Formation of Pseudostellaria heterophylla

The quality of *P. heterophylla* primarily depends on the developmental status of its tuberous roots and the accumulation of three major bioactive components: saponins, cyclopeptides, and polysaccharides. This regulation relies on the synergistic interaction between phytohormones such as indole-3-acetic acid (IAA), abscisic acid (ABA), gibberellin (GA_3_), and paclobutrazol (PBZ), as well as key gene families, including NF-Y, GST, GA2ox, and PCY. Each regulatory factor forms a complex and ordered regulatory network through tissue-specific expression, signal crosstalk, and precise regulation of target genes.

The synergistic effect of IAA and ABA is the core driver in the initial stage of tuberous root enlargement, and the balance between IAA synthesis and metabolism directly affects tuber diameter. Exogenous 20 mg/L PBZ upregulates the IAA synthesis gene *YUCCA* (Unigene49937) and the *GH3* family metabolic gene Unigene37777 at 10, 20, and 30 days post-treatment, increasing endogenous IAA content by 18.2%, 12.5%, and 9.8% compared to the control, respectively. During this period, tuber diameter increases significantly, and tuber morphology becomes thicker. After 40 days, IAA content decreases, and tuber diameter growth slows down and stabilizes. Exogenous 150 mg/L GA_3_ generally promotes IAA accumulation (with only a 5.6% decrease at 20 days) but inhibits *YUCCA* expression; in the *GH3* family, Unigene43146 and Unigene43412 are inhibited by PBZ, while Unigene37777, Unigene43146, and Unigene43412 are all induced by GA_3_. This differential expression shows tissue specificity: *YUCCA* is enriched in flowers and leaves, while GH3 family genes are highly expressed in the phloem and xylem of tuberous roots, suggesting IAA needs to be synthesized in aerial parts and transported to tuberous roots to regulate the morphological transition of tubers from slender to short and thick [49].

ABA further promotes the quality formation of tuberous roots by regulating cell structure and substance accumulation. Integrated transcriptomic and proteomic analyses show that 15 mg/L ABA can upregulate the auxin signaling gene *PhIAA1* and jasmonic acid (JA) synthesis genes *PhLOX1/2/3/5/7*, while downregulating the cell wall-loosening gene *PhXTH1* and the starch synthesis gene *PhSusy3*. These changes increase the transverse width of xylem cells in tuberous roots by 28.6% and the number of starch granules by 35.2%, further increasing tuber diameter and making the tuber morphology more short, thick, and plump. Transcription factors such as *PhMYB10* and *PhbZIP2* can mediate ABA signaling and optimize tuber structure by regulating genes like *PhABC-transporter7* (involved in lignin transport) [50].

Notably, ABA also modulates cytokinin (CTK) metabolism to coordinate tuber development: it induces the expression of *PhCKX* (a key gene for CTK degradation, encoding a membrane protein with FAD and CTK binding domains) in the middle stage of tuber growth, which in turn adjusts endogenous CTK levels—this regulatory link between ABA and CTK further supports the balanced development of tuberous roots [51].

GA_3_ and PBZ determine the final morphology of tuberous roots through antagonistic effects. Treatment with GA_3_ (75–150 mg/L) for 60 days significantly increases stem length and above-ground biomass, but significantly reduces tuber diameter and underground biomass, leading to a slender tuber morphology. Simultaneously, GA_3_ increases the contents of zeatin riboside (ZR), GA_3_, and IAA in the xylem of tuberous roots, while decreasing the contents of ABA and methyl jasmonate (MeJA). In contrast, PBZ (20 mg/L) reverses these effects: it significantly reduces stem length, decreases xylem radius, and significantly increases tuber diameter, restoring the tuber to a short and thick morphology [52]. This antagonism is closely related to the *GA2ox* gene family: *PhGA2ox1* (Class I, 981 bp coding sequence) can inactivate C_19_-GAs such as GA_1_ and GA_4_ and is stably expressed in all tissues; *PhGA2ox8* (Class III, 1056 bp coding sequence) specifically inactivates C_20_-GAs such as GA_12_ and GA_53_ and is highly expressed in the xylem of tuberous roots (RPKM = 12.7), which is 5.3 times its expression level in leaves. Both genes jointly participate in the local inactivation of GA_3_ to maintain the balanced development of short and thick tubers [53].

The NF-Y gene family also plays a bridging role in tuberous root development and stress response. Among the 24 members (9 *PhNF-YA*, 10 *PhNF-YB*, and 5 *PhNF-YC*), *PhNF-YA4* and other members are highly expressed in leaves, *PhNF-YA3* is enriched in the tuber cortex, and the *PhNF-YB3/8* genes are specifically expressed in the tuber xylem. Eleven members respond to drought stress, and most respond to changes in sucrose concentration, suggesting that they can mediate the interaction between water/sucrose signals and phytohormones, and regulate the development process of tubers from initial enlargement to the formation of a stable short and thick morphology [54].

In the regulation of bioactive component accumulation, phytohormones and gene families exhibit significant selective effects. Saponin synthesis is inversely regulated by GA_3_ and ABA: long-term treatment with GA_3_ (150 mg/L) upregulates the squalene epoxidase gene *SE1* and the isopentenyl pyrophosphate isomerase gene *IPPI*, with an extremely significant positive correlation between the two, increasing saponin content by 45.6% compared to the control. In contrast, ABA (15 mg/L) inhibits saponin synthesis by downregulating the 3-hydroxy-3-methylglutaryl-CoA synthase gene *HMGS* and the mevalonate diphosphate decarboxylase gene *MDD*. Additionally, the key enzyme gene for the final step of saponin synthesis, *β-amyrin 28-oxidase* (β-A28O), is also regulated by both hormones: GA_3_ upregulates it by 1.8 times, while ABA downregulates it by 1.6 times [55]. Notably, for another key bioactive component (polysaccharides), its synthesis-related cellulose synthase-like gene *PhCslG* responds to GA_3_ concentration: low-to-medium GA_3_ (20–75 mg/L) promotes *PhCslG* expression and long-term polysaccharide accumulation, while high GA_3_ (150 mg/L) suppresses both, and GA_3_ inhibitor PBZ (20 mg/L) enhances polysaccharide content [56].

Cyclopeptide synthesis depends on the key peptide cyclase *PhPCY3*. This gene, through the critical sites N500 and S502 in its prolyl oligopeptidase domain (mutation reduces the content of heterophyllin B (HB) by 62.3–78.5%), specifically catalyzes the cleavage of the C-terminal flanking sequence of linear precursor peptides (e.g., PreHB [14,15,16,17,18,19,20,21,22,23,24,25,26,27,28,29,30,31,32,33,34,35]) and the cyclization of core peptides, forming known cyclopeptides such as heterophyllin B (HB) and pseudostellarin E/F/G. More than 100 precursor peptide genes mined from the transcriptomes of five cultivars can also be used to biosynthesize four novel orbitides (e.g., cyclo-[LDGPPPYF] and cyclo-[WGSSTPHT]) via a Nicotiana benthamiana heterologous expression platform [57].

Polysaccharide accumulation is related to IAA concentration and the GST gene family: when IAA content exceeds a certain threshold, it may affect polysaccharide synthesis-related genes, while the accumulation of endogenous IAA itself is regulated by exogenous PBZ and GA_3_ during the development of *P. heterophylla* tuberous roots [49]. Among the GST family, *PhGSTZ3* and *PhGSTU6* show a significant positive correlation with polysaccharide accumulation; *PhGSTU8* can also be upregulated under specific conditions to promote polysaccharide accumulation. Meanwhile, the GST family exhibits selectivity in regulating bioactive components: *PhGSTL1* and *PhEF1Bγ4* are positively correlated with HB accumulation, while *PhGSTF1* is negatively correlated [58]. Additionally, exogenous regulators like 6-BA, which enhances photosynthetic performance and antioxidant activity in the late growth stage of *P. heterophylla*, can indirectly create a favorable physiological environment for the accumulation of bioactive components such as polysaccharides [59].

**Figure 5:**
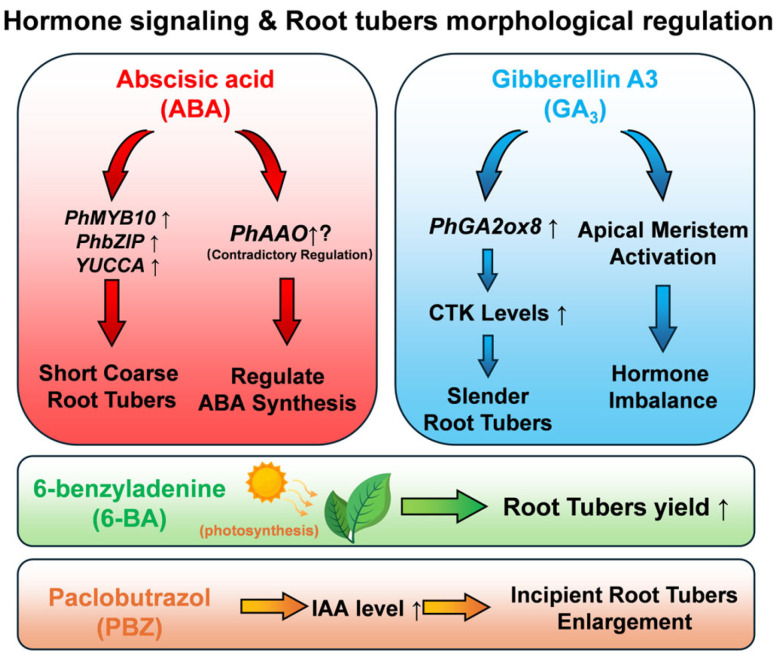
Hormone signaling pathway diagram of morphological regulation of *Pseudostellaria heterophylla* root block.

In addition, the quality regulation of *P. heterophylla* involves complex signal crosstalk and mechanistic details: the key ABA synthesis gene *PhAAO* (full length, 6773 bp, containing a molybdenum–flavin-enzyme conserved domain) is upregulated by both exogenous ABA and fluridone [60] (Figure 5). MicroRNAs (miRNAs) also participate in hormone signal integration: miR171 targets genes of the SCL6 family related to hormone signaling, and miR393 targets the auxin signal-related gene *TIR1*; however, the tissue-specific expression of these miRNAs in the tuber phloem/xylem requires further verification via in situ hybridization [61]. These regulatory details collectively ensure the coordinated optimization of tuberous root development and bioactive component accumulation in *P. heterophylla*, forming the core mechanism of its quality formation.

In the future, it is necessary to further integrate the tuberous root regulatory mechanism with cultivation links such as propagation, soil management, continuous cropping, and stress resistance, and use techniques like gene editing and precise hormone application to achieve the comprehensive goal of “excellent morphology, high component content, and strong stress resistance” for *P. heterophylla* tubers, promoting the sustainable development of its industry.

## 4. Discussion

*Pseudostellaria heterophylla* (Miq.) Pax ex Pax et Hoffm. (common name: false starwort; medicinal name: Taizishen), a dual-purpose herb with growing pharmacological importance, has advanced in phytochemistry and cultivation research. Yet critical gaps remain in connecting its bioactive components to molecular mechanisms, addressing cultivation challenges, and optimizing sustainable use—gaps amplified by disconnected research across these areas. While 19 cyclic peptides, 10 polysaccharides, and 6 saponins have been characterized, the research on its pharmacological effects is fragmented. For example, heterophyllin B exhibits neuroprotective effects in Alzheimer’s disease models but lacks direct evidence of modulating the BACE1 enzyme; polysaccharides show TLR4-mediated immune regulation, yet their synergistic interactions with cyclic peptides remain unstudied. This fragmentation not only delays clinical translation but also undermines the design of cultivation strategies for targeted component accumulation; it not only delays clinical transformation, but also restricts the design of cultivation strategies for “targeted accumulation of active ingredients”, directly affecting the reproducibility of mechanism studies.

A core challenge lies in integrating biocultivation solutions with the mitigation of continuous cropping obstacles—two interconnected issues that directly shape both yield and active component quality. Continuous cropping drives soil acidification and pathogen proliferation, while phenolic acid accumulation further disrupts rhizosphere balance by promoting pathogen colonization and inhibiting beneficial *Bacillus pumilus*. Biocultivation strategies offer viable solutions: the “flooding + microbial fertilizer” treatment boosts the abundance of beneficial *Burkholderia* and reduces phenolic acid content, restoring heterophyllin B and saponin levels to those of non-cropped soils; rhizosphere microbiome engineering upregulates *PhGSTU8*, enhancing stress tolerance and polysaccharide synthesis. Crucially, this connection is bidirectional: resolving continuous cropping obstacles provides consistent samples for mechanistic studies of active components, while understanding these mechanisms guides the selection of microbial consortia that enhance targeted components, forming a mutually reinforcing cycle. However, current biocultivation approaches lack precision—they do not account for genotype-specific responses or dynamic environmental changes, highlighting the need to link soil diagnostics with component-driven cultivation goals.

While research on phytohormones has clarified the antagonistic effect of ABA-GA_3_ on tuberous root morphology, it has oversimplified the connections between this effect and environmental factors, as well as spatiotemporal dynamics. The cloned *PhGA2ox8* (a gibberellin-inactivating enzyme) is highly expressed in the xylem of tuberous roots; it promotes tuberous root enlargement by catalyzing the inactivation of *GA_12_/GA_53_* [53]. However, soil acidification, caused by continuous cropping, downregulates the expression of *PhGA2ox8*, disrupting gibberellin homeostasis and inhibiting tuberous root development [15]. Exogenous hormone treatments often overlook stage-specific responses during development: GA_3_ can upregulate the expression of the *WOX5* gene in root tips, which may inhibit tuberous root elongation, yet targeted validation using *pWOX5*-driven reporter genes is lacking [52]. This disconnect between hormones, the environment, and bioactive components limits the possibility of achieving simultaneous improvements in yield and quality through precise hormone regulation.

In conclusion, advancing *P. heterophylla* research requires three overarching priorities. First, integrate metabolic and regulatory networks—linking environmental cues (soil pH and microbial communities), hormone signals (ABA-GA_3_ balance, *PhGA2ox8*, and *PhNF-Y* genes), and active component synthesis (cyclic peptides, saponins, and polysaccharides)—to address current fragmentation in mechanistic understanding. Second, advance precision biocultivation: combine IoT-based soil monitoring, rhizosphere microbiome engineering, and understory farming to mitigate continuous cropping obstacles while targeting the accumulation of key active components. Third, establish translational pipelines that bridge phytochemical discoveries with clinical validation, ensuring research translates to both healthcare applications and sustainable agricultural practices. These priorities, rooted in the inherent connections between environmental factors, hormonal regulation, and active components, are essential to transforming *P. heterophylla* from a traditional tonic into a science-driven, sustainable resource.

## Figures and Tables

**Table 1:** Main components of *Pseudostellaria heterophylla*.

Category	Specific Components
Polysaccharides	PF40, PF60, PF90, PHP, PHP-A, PHP-B, H-1-2, HP_h–1–1_, 0.5MSC-F, PHP90
Cyclopeptides	Heterophyllin H, heterophyllin J, pseudostellarin A, pseudostellarin L, heterophyllin D, heterophyllin F, pseudostellarin K, heterophyllin A, heterophyllin C, heterophyllin G, pseudostellarin D, heterophyllin B, pseudostellarin B, pseudostellarin C, pseudostellarin F, pseudostellarin G, pseudostellarin H, pseudostellarin E, heterophyllin E
Organic acids and their derivatives	Dihydroferulic acid, ferulic acid, methyl ferulate, methyl dihydroferulate, dimethyl phthalate, dibutyl phthalate, succinic acid, salicylic acid, pyrrole-2-carboxylic acid, 3-furancarboxylic acid, 3-fumuy l pyrrole-2-carboxylate, benzoic acid, octadecatrinoic acid, linolenic acid, palmitic acid, linoleic acid, stearic acid, tetracosanoic acid, tripalmitin
Saponins and sapogenins	Taraxerol, taraxerol acetate, ursolic acid, β-sitosterol, Pseudotellarinoside A, acutifoliside D, β-Sitosterol-3-O-β-D-glucoside, β-Sitosterol-3-O-β-D-glucoside-6′-O-palmitate, Δ^7^-stigmastenol-3-O-β-D-glucopyranoside, α-Spinasterol-3-O-β-D-glucopyranosides

**Table 2:** Summary of research methods for chemical constituents in *Pseudostellaria heterophylla*.

Active Component Type	Experimental Organ	Key Experimental Methods	Literature Source	Remarks
Cyclic peptides	Dried root tubers	Silica gel column chromatography, MS, NMR	[16,18,19]	19 types (heterophyllins A–H, J; pseudostellatins A–H, K, L)
Sapogenins	Dried root tubers	Silica gel column chromatography, ESI-MS, NMR	[17]	4 types
Saponins	Dried root tubers	Silica gel column chromatography, ESI-MS, NMR	[17]	6 types
Polysaccharides	Dried root tubers	DEAE-52 ion-exchange chromatography, Sephadex S-300 gel chromatography, GC, NMR, HPGPC	[16,20]	10 types
Oligosaccharides	Dried root tubers	Pressurized liquid extraction (PLE), UPLC-CAD, UPLC-QTOF MS	[16,17,20]	7 types
Volatile oils	Dried root tubers	Supercritical CO_2_ extraction, HS-SPME, GC-MS	[21]	–
Sterols	Dried root tubers	Supercritical CO_2_ extraction, HS-SPME, GC-MS	[21]	Δ^7^-stigmasten-3β-ol, Δ^7^-stigmasten-3-O-β-D-glucopyranoside, etc.
Other compounds	Dried root tubers	D101 macroporous resin, Sephadex LH-20, HR-ESI-MS, NMR, ECD	[21]	Pseudostellarin A, essential amino acids, etc.

**Table 3:** Pharmacological activities of main components of *Pseudostellaria heterophylla*.

Experimental Model	Activity Results	Literature Source
Normal/immunocompromised mice	The water-soluble bioactive components of the superfine powder suspension *P. heterophylla* enhance immune function by upregulating the expression of interleukin-2 (IL-2) and interferon-γ (IFN-γ), and reshape the gut microbiota.	[22]
Macrophage RAW264.7	Hydrophilic polysaccharides (under ultrasonic treatment) enhanced cell proliferation and activity, improved antioxidant and immune functions	[22]
Mouse OVA immune model	PHP promoted splenic lymphocyte proliferation; enhanced NK cell cytotoxicity; and upregulated IL-4, IL-10, and IFN-γ transcription	[23]
Mouse/porcine models	PHP increased Prevotella copri abundance, elevated TDCA levels, and regulated immunity via IL-6/JAK1/STAT3 and RelA pathways	[24,25,26,27]
AD mouse model	Heterophyllin B alleviated neuroinflammation, reduced Aβ/Tau, and regulated the spleen–gut–brain axis	[29,30]
BLM-induced PF mouse model	Heterophyllin B inhibited fibroblast transformation and ECM deposition via TGF-Smad2/3 and AMPK-STING pathways	[31]
GC cells (AGS, HGC-27)	Luteolin and acacetin bound to TP53, promoted its phosphorylation, and inhibited cell viability via ROS- and P53-related pathways	[13]
DLBCL cells	Active components affected cell apoptosis by targeting CASP3	[6]
AD network pharmacology model	Heterophyllin B interacted with 5 key targets (ALB, MMP9, Src, EGFR, and MMP2)	[30]

## Data Availability

No datasets were generated or analyzed during the current study.
